# Correction: The emerging role of medical foods and therapeutic potential of medical food-derived exosomes

**DOI:** 10.1039/d3na90122j

**Published:** 2024-01-15

**Authors:** Jin-Young Hur, SeonHyung Lee, Woo-Ri Shin, Yang-Hoon Kim, Ji-Young Ahn

**Affiliations:** a Department of Microbiology, Chungbuk National University 1 Chungdae-Ro, Seowon-Gu Cheongju 28644 South Korea jyahn@chungbuk.ac.kr kyh@chungbuk.ac.kr +82-43-264-9600 +82-43-261-2301; b Department of Bioengineering, University of Pennsylvania 210 S 33rd St Philadelphia PA 19104 USA

## Abstract

Correction for ‘The emerging role of medical foods and therapeutic potential of medical food-derived exosomes’ by Jin-Young Hur *et al.*, *Nanoscale Adv.*, 2024, https://doi.org/10.1039/d3na00649b.

The authors regret that the affiliation of the author SeonHyung Lee was incorrectly listed as the University of Pennsylvania in the original manuscript. The correct affiliation is listed above.

The Royal Society of Chemistry apologises for these errors and any consequent inconvenience to authors and readers.

## Supplementary Material

